# Flora diversity survey and establishment of a plant DNA barcode database of Lomas ecosystems in Peru

**DOI:** 10.1038/s41597-023-02206-y

**Published:** 2023-05-19

**Authors:** Feng Song, Yun-Fei Deng, Hai-Fei Yan, Zhe-Li Lin, Amalia Delgado, Huber Trinidad, Paúl Gonzales-Arce, Sebastián Riva, Asunción Cano-Echevarría, Elmer Ramos, Yaquelin Pamela Aroni, Soledad Rivera, Mónica Arakaki, Xue-Jun Ge

**Affiliations:** 1 Key Laboratory of Plant Resources Conservation and Sustainable Utilization, South China Botanical Garden, Chinese Academy of Sciences, Guangzhou, 510650 China; 2 Henry Fok College of Biology and Agriculture, Shaoguan University, Shaoguan, 512005 China; 3 División Botánica, Museo de Historia Natural, Universidad Nacional Mayor de San Marcos, Av. Arenales 1256, Lima, 11 Perú

**Keywords:** Plant molecular biology, Taxonomy

## Abstract

Lomas formations or “fog oases” are islands of vegetation in the desert belt of the west coast of South America, with a unique vegetation composition among the world’s deserts. However, plant diversity and conservation studies have long been neglected, and there exists a severe gap in plant DNA sequence information. To address the lack of DNA information, we conducted field collections and laboratory DNA sequencing to establish a DNA barcode reference library of Lomas plants from Peru. This database provides 1,207 plant specimens and 3,129 DNA barcodes data corresponding with collections from 16 Lomas locations in Peru, during 2017 and 2018. This database will facilitate both rapid species identification and basic studies on plant diversity, thereby enhancing our understanding of Lomas flora’s composition and temporal variation, and providing valuable resources for conserving plant diversity and maintaining the stability of the fragile Lomas ecosystems.

## Background & Summary

Along the Pacific coast from 5°S (northern Peru) to 30°S (northern Chile), a narrow arid belt, of nearly 3,000 km long, is formed at the foot of the Andes^1^. This belt is typified by a tropical desert climate, with annual precipitation of less than 50 mm (arid) or 5 mm (hyper-arid), making it one of the driest areas in the world. Despite the drought, “fog oasis” like islands with lush vegetation and flowers appear in the desert and are known as “blooming deserts”^2,3^. There is a tenuous balance between Pacific Ocean currents, onshore winds, the height of the Andes mountains, and cyclic El Niño events that shape such particular geographic and unique climatic conditions^4–6^. From July to November, ocean-generated fog is intercepted by isolated mountains or steep coastal slopes, starting at sea level and extending to around 1,000 meters asl. This is the main water source sustaining plant communities from arid and hyper-arid zones, locally known as Lomas formations (Peru) or “fog oases” (Chile)^4,6,7^.

Lomas ecosystems, due to their seasonal and ephemeral nature, are one of the most climate-responsive ecosystems on Earth. Human activities, such as urban sprawl, mining, off-road vehicle, dumping, overgrazing, and the introduction of invasive plants and animals, have had a significant impact on them^4,5,8^. More than 58% of the Peruvian population lives in the narrow arid belt of the Pacific coast and relies on the Lomas for their livelihood^5^. However, only 3.3% of Peruvian Lomas are currently included in formally protected areas^5^.

The importance of Lomas plant diversity has been overlooked in comparison to the adjacent Andean-Amazonian World Biodiversity Center^9^. This diversity is both unique and fragile, with more than 40% of the species, within individual Lomas communities, being endemic, such as species in genera *Calceolaria*, *Mathewsia*, *Nolana*, *Palaua*, *Tiquilia*, *Weberbauerella*, etc.^1^. The Lomas are a valuable genetic resource, containing wild relatives of many crops (e.g., Andean potato, tomato, and papaya), as well as medicinal plants (e.g., *Nasa urens*, *Alternanthera halimifolia*), and fuel plants (e.g., *Caesalpinia spinosa*, *Vachellia macracantha*), among others^8^.

The composition of each Lomas plant community is highly diverse, resulting from a combination of climate, geography, and the eco-physiology^1^. Investigating the formation of plant diversity in Lomas is a challenge, because of annual inconsistencies in desert precipitation, and often very high inter-annual climate variability^10^. The number of species in the Lomas flora fluctuates dynamically^11^, with rare species potentially appearing only during or after El Niño events, and then not being found for several years^4^. Several regional floristic checklists in Peru have been published, for example, for La Libertad^12^, Ancash^13^, Lima^14–16^, Ica^17,18^ and Arequipa^19^.

However, many taxa remain unnamed at species or genus levels. This transient and heterogeneous appearance of Lomas vegetation has limited the opportunities for plant collections or occurrence records^1,20^, making species taxonomic revision in the Lomas flora difficult. The number of specimens registered for Peruvian Lomas is limited. The Field Museum in Chicago has the most comprehensive collection of Lomas plants from Peru and Chile, with ca. 2,800 specimens from Peruvian Lomas retrievable from its online botanical collections database (https://collections-botany.fieldmuseum.org/project/6657), covering 723 species from 369 genera in 92 families. Additionally, about 3,800 Lomas plant specimens of 438 species from 253 genera in 79 families can be retrieved from the Global Biodiversity Information Facility (GBIF, https://www.gbif.org/) database.

DNA barcoding techniques and specific reference libraries offer new opportunities for rapid, accurate, and automated species identification. They compensate for the disadvantages of morphology-based taxonomic methods which are time-consuming, specialized, and the identification results are subject to the integrity and life stage of the specimen. Consequently, this decreases the efficiency of field species survey and identification processes^21–26^. To accelerate the species investigation and conservation process in the Lomas flora, we (1) made a catalogue of the flora of Peruvian Lomas based on historical plant species lists from the published literature, and online specimen databases; (2) conducted a species survey and collection in Peruvian Lomas formations and established a plant DNA barcode reference library. Voucher specimen have been deposited in scientific collections. Images and DNA barcode sequences has been uploaded to the web-based information workbench, called the Barcode of Life Data System (BOLD)^27^. A publicly available DNA reference library of Lomas plants will provide a valuable resources to all scholars interested in Lomas vegetation. Although it is a small-scale focus, the diversity of unique taxa will be expected to have broad utility in plant biogeography, biodiversity conservation, environmental DNA detection, biological invasion detection, and the food industry.

## Methods

### Generation of the flora list for Lomas

The floristic list of Lomas was obtained from previously published studies (see Supplementary File 1: Reference) and specimen records from the Field Museum’s online Botanical Collections Database (https://collections-botany.fieldmuseum.org/project/6657). A total of 4,653 records were retrieved for the Peruvian Lomas, each of which was retained in Supplementary File 1 (Literature_record: 1,816; Specimen_record: 2,837), with the specific source in the ‘REFERENCE’ column (as of March 2023). Species names and taxonomic authorities were verified using the ‘status’ function of the R package ‘plantlist’ version 0.8.0^28^, and the World Checklist of Vascular Plants (WCVP)^29^. The original species names and the updated species names are presented in Supplementary File 1, with different columns for ‘ORIGINAL_TAXON_NAME’ and ‘ACCEPTED_SPECIES_NAME’, respectively. The family classification of angiosperms follows APG IV^30^ and its updated website APW (http://www.mobot.org/MOBOT/Research/APweb), gymnosperms follows Yang *et al*. in 2022^31^, and ferns and lycopods follows Christenhusz & Chase^32^.

### Specimen collection and identification

**Fig. 1:**
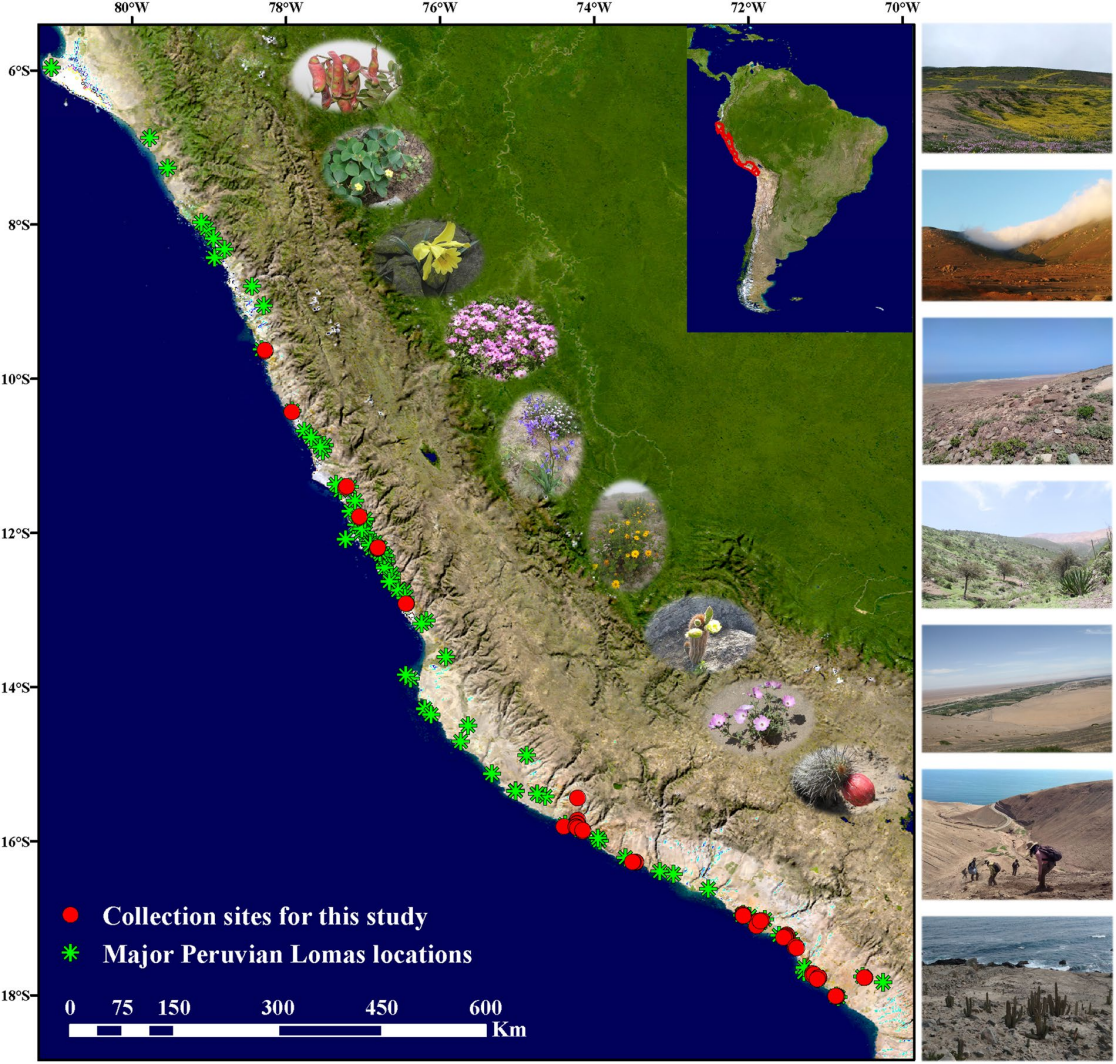
Map of Lomas formation sites in Peru. Red dots represent collection sites for this study. Green symbols indicate the main Lomas sites in Peru from Moat *et al*.^5^ and Ruhm *et al*.^47^. The map was generated using ArcGIS 10.1.

In 2017 and 2018, two field trips were organized to collect specimens in the arid belt of the west coast of Peru, ranging from 9.37°S to 18.00°S latitude, 70.29°W to 78.17°W longitude, and 20 to 1,270 meters elevation. The collection campaign extended from September to November during the wet season in Peru, from north to south, with 1,207 specimens collected from 16 main Lomas locations (Table 1; Fig. 1). The environments varied from place to place (see Table 1 for details). Due to the widespread climatic variations of El Niño–Southern Oscillation (ENSO), Lomas vegetation is characterized by its transient and disparate nature of occurrence, which limits the opportunities for collection and plant occurrence records^4,20,33^. For each sampling locality, we: (1) collected all the plant species found; (2) recorded GPS spatial location, elevation, and habitat characteristics; (3) preserved plant leaf tissue in silica gel; (4) recorded the key characteristics of each specimen and made a preliminary identification of them. All collections were conducted in compliance with national and local regulations under permits N° 310-2017-SERFOR/DGGSPFFS and N° 429-2018-MINAGRI-SERFOR-DGGSPFFS granted by the Peruvian National Forestry and Fauna Service. All the specimens were deposited in the herbarium of South China Botanical Garden (IBSC) and Herbario San Marcos (USM) of the Natural History Museum (UNMSM) in Lima. Following the inclusion in the scientific collections, the specimens were further examined and identified by professional taxonomists from the Herbario San Marcos (USM) to confirm the initial identification made in the field.

**Table 1:** Detail of botanical specimen collection in Peruvian Lomas.

Department	Locations	Sampling	GPS Coordinates	Altitude	Habitat	Habit
Ancash	Casma Province, Casma District, Lomas de Mongón	67	9°37′S-9°38′S, 78°16′W-78°17′W	380–600 m	Alternatives: slope, ravine, sandy soil, rock, grassland, swallet, stones, hill, scree, thicket, wet place, flowing beach, roadside, streamside, epiphyte on tree	Alternatives: herb, sprawling herb, shrub, subshrub, herbaceous vine, vine, tree
	Bolognesi Province, Lomas de Lupín	16	10°25′S, 77°55′W	340–365 m		
Lima	Huaral Province, Huaral Dictrict, Lomas de Iguanil	77	11°24′S, 77°13′W	320–660 m		
	Lima Province, Carabayllo District, Lomas de Carabayllo	31	11°47′S-11°48′S, 77°02′W	610–690 m		
	Cañete Province, Lomas de Quilmaná	48	12°55′S, 76°26′W-76°27′W	330–470 m		
	Lima Province, Pachacámac District, Santuario del Amancay	67	12°11′S-12°12′S, 76°48′W-76°49′W	320–600 m		
Arequipa	Islay Province, Islay District, Jesús	87	17°13′S-17°15′S, 71°30′W-71°32′W	160–770 m		
	Caravelí Province, Atico District	82	16°15′S-16°16′S, 73°28′W-73°30′W	55–210 m		
	Caravelí Province, Atiquipa District, Lomas de Atiquipa	226	15°43′S-15°49′S, 74°21′W-74°24′W	20–1270 m		
	Caravelí Province, Chala District, Lomas de Cháparra	58	15°51′S-15°52′S, 74°08′W	260–740 m		
	Islay Province, Mejía District, Lomas de Mejía	165	17°03′S-17°05′S, 71°52′W-71°53′W	250–920 m		
	Islay Province, Lomas de Yuta	66	16°56′S-16°58′S, 72°04′W	420–835 m		
Moquegua	Ilo Province, Pacocha District, Pacocha	33	17°22′S-17°23′S, 71°22′W-71°23′W	180–630 m		
	Ilo Province, Ilo District, Ilo	41	17°42′S-17°44′S, 71°08′W-71°10′W	436–695 m		
Tacna	Tacna Province, Sama District, Sama	84	17°46′S-18°00′S, 70°29′W-70°52 W	120–785 m		
	Jorge Basadre Province, Ite District, Tacahuay	59	17°46′S, 71°05′W	430–900 m		
Total	16	1207

### DNA barcode generation

Fresh leaves were collected and stored in silica gel during fieldwork. Total genomic DNA was extracted using the CTAB method^34^. We used two plastid protein-coding DNA regions and a ribosomal DNA region, corresponding to the most widely used plant DNA barcodes^35,36^: the ribulose-bisphosphate/carboxylase Large-subunit gene (*rbcL*), the maturase-K gene (*matK*), and the internal transcribed spacer 2 (ITS2). Sequencing was performed using universal DNA barcode primers for *rbcL*^37^ (*rbcLa*_For: ATGTCACCACAAACAGAGACTAAAGC; *rbcLa*_Rev: GTAAAATCAAGTCCACCRCG), *matK* (K. J. Kim, unpublished, Kim-3F: CGTACAGTACTTTTGTGTTTACGAG; Kim-1R: ACCCAGTCCATCTGGAAATCTTGGTTC), ITS2^38^ (ITS2_S2F: ATGCGATACTTGGTGTGAAT; ITS2_S3R: GACGCTTCTCCAGACTACAAT). The PCR reaction mixture was made up of 25 μl (2.5 μl of 10 × PCR buffer (Tris-HCl, 100 mM; KCl, 500 mM; MgCl_2_, 15 mM), primer pairs 0.5 μl (10 μM) each, 2.0 μl of dNTPs (2.5 µM), 0.5 μl of DNA template (about 20~30 ng), 0.2 μl of rTaq polymerase (5 U µl^−1^), and 18.8 μl of ddH_2_O. The PCR amplification was performed under the following conditions: 3 min at 94 °C; 35 cycles of 30 secs at 94 °C, 45 secs at 50 °C for *rbcL*, 48 °C for *matK*, and 55 °C for ITS2, 1 min at 72 °C; and 10 min at 72 °C. All PCR products were visualized on a 1.0% agarose gel and sequenced on an ABI3730 DNA analyzer. ITS2 and *matK* DNA fragments were sequenced in bi-direction to ensure the accuracy of sequence data, and *rbcL* was sequenced in the forward direction. Raw sequences were trimmed and forward and reverse reads were assembled in Geneious 11.0.2^39^. The newly obtained sequences were initially verified by BLASTn method: All sequences were confirmed as the correct target fragments using BLASTn search of the GenBank database (https://blast.ncbi.nlm.nih.gov/Blast.c-gi). Considering the absence of some species in the GenBank database, the target fragments with the highest bit-scores for sequence accessions from the same genus or family were regarded as reliable^40,41^. Ultimately, 1,157 samples had at least one sequence matching them, and 858 samples had sequence data for all three DNA barcode sequences (*rbcL*, *matK* and ITS2).

### Data verification

The accuracy of DNA barcodes is tested by (1) The barcoding gap method: Comparing the K2P (Kimura 2 Parameter) intra- and inter-specific distances for each barcode, where distances were calculated by MEGA-X^42^, and species were identified to have barcode gaps when the maximum intra-specific distance is lower than the minimum inter-specific distance^43^; (2) The Best Close Match (BCM) method: Identification efficiency of each DNA barcode was calculated using taxonDNA^44^; (3) The tree-based method: The sequence obtained was considered correctly identified if all sequences from the same species, genus or family form a monophyletic clade in a phylogenetic tree. Briefly, the obtained sequences were first aligned using MAFFT v7.471^45^ and manually adjusted in Genious 11.0.2^39^. All obtained *rbcL* and *matK* sequences were aligned simultaneously, while ITS2 sequences were aligned by order level. The *rbcL*, *matK*, and ITS2 alignments were then combined to create a super-matrix alignment (RMI2). Maximum-likelihood (ML) trees were generated using RAxML-HPC2 (8.2.12)^46^ with the GTRGAMMA model, and the support for individual nodes in the phylogenetic tree was assessed with 1,000 bootstrap replicates. The sequence obtained was considered correctly identified if all sequences from the same species, genus, or family form a monophyletic clade with bootstrap support (BS) of not less than 50%.

## Data Records

To date, 4,653 Peruvian Lomas plant records have been accumulated and summarized in Supplementary File 1. Excluding 276 records not assigned to the species level, there are 1,092 vascular plant species belonging to 468 genera and 96 families in the Peruvian Lomas flora. The dominant families are Solanaceae (17 genera/116 species), Asteraceae (52/103), Poaceae (43/96), Fabaceae (37/79), and Malvaceae (24/67) (Table 2). Each record in Supplementary File 1 represents a species in the literature and specimen databases, with associated information, including (1) Literature_record: ORIGINAL_TAXON_NAME, ACCEPTED_SPECIES_NAME, ACCEPTED_AUTHOR, TAXONOMIC_STATUS, GENUS, FAMILY (APG IV), ORDER, GROUP, REFERENCE, NATIVE_VS_CULTIVATED; (2) Specimen_record: ORIGINAL_TAXON_NAME, ACCEPTED_SPECIES_NAME, ACCEPTED_AUTHOR, TAXONOMIC_STATUS, GENUS, FAMILY (APG IV), ORDER, GROUP, REFERENCE, Collector, IRN, occurrence ID, Catalog Number.

**Table 2:** Families ranked by number of species for Lomas plants in Peru.

Recorded lists			Field collection		
Family	Genera	Species	Family	Genera	Species
Solanaceae	17	116	Asteraceae	28	34
Asteraceae	52	103	Solanaceae	10	32
Poaceae	43	96	Malvaceae	9	23
Fabaceae	37	79	Poaceae	15	21
Malvaceae	24	67	Boraginaceae	7	15
Cactaceae	21	45	Fabaceae	11	13
Boraginaceae	9	35	Lamiaceae	6	11
Brassicaceae	14	31	Amaranthaceae	4	10
Convolvulaceae	9	30	Euphorbiaceae	2	7
Amaranthaceae	9	27	Brassicaceae	5	6
Euphorbiaceae	7	25	Amaryllidaceae	4	6
Lamiaceae	10	22	Oxalidaceae	1	6
Caryophyllaceae	7	18	Convolvulaceae	5	5
Cyperaceae	5	17	Verbenaceae	5	5
Apiaceae	10	14	Pteridaceae	3	5
Verbenaceae	10	16	Cactaceae	4	5
Nyctaginaceae	6	16	Caryophyllaceae	4	4
Bromeliaceae	4	16	Loasaceae	3	4
Amaryllidaceae	8	12	Montiaceae	2	3
Acanthaceae	7	15	Geraniaceae	2	4
Plantaginaceae	7	12	Cyperaceae	2	3
Cucurbitaceae	7	12	Apiaceae	3	3
Loasaceae	5	12	Bromeliaceae	2	3
Oxalidaceae	1	12	Plantaginaceae	3	3
Rubiaceae	7	11			
Other	132	233	Other	52	58
Total	468	1092	Total	192	289

The distribution of main Lomas^5,47^ and the Lomas visited for this work are shown in Fig. 1. After two years of surveys, 1,207 specimens were collected, of which, 870 were identified to species level (289 species from 192 genera in 67 families). The remaining 337 specimens were identified to genus level (336 individuals in 134 genera) and one to family level (Supplementary File 2), due to missing critical features based on morphological identification, the lack of reference barcode data, barcode sequencing failures or insufficient barcode resolution. This yielded a total of 238 genera from 78 families (Table 3). The dominant families are Asteraceae (28/34, genera/species), Solanaceae (10/32), Malvaceae (9/23), Poaceae (15/21), Boraginaceae (7/15), Fabaceae (11/13), Lamiaceae (6/11), and Amaranthaceae (4/10) (Table 2). The 38 taxa (33 genera and 22 families) are new records (Supplementary File 2) compared with the catalogue of Peruvian Lomas flora in this study.

**Table 3:** Summary of the Core DNA barcode for Lomas plants in Peru.

Sequence information	Collected data	DNA barcodes							
		*rbcL*	%	*matK*	%	ITS2	%	RMI2	%
Number of sampled species	289	276	96%	253	88%	246	85%	225	78%
Number of sampled genera (including spp.)	238	233	98%	215	90%	203	85%	190	80%
Number of sampled families	78	76	97%	64	82%	64	82%	57	73%
Number of sampled individuals	1207	1127	93%	1038	86%	964	80%	858	71%

**Fig. 2:**
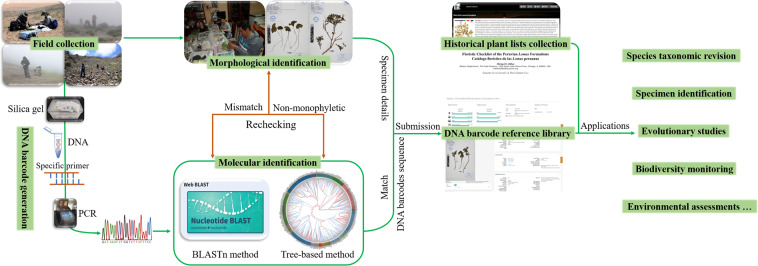
Process of data generation.

We generated 3,129 plant DNA barcodes for 1,157 collected specimens, including 1,127 *rbcL*, 1,038 *matK*, and 964 ITS2 sequences (Table 3), with sequencing success rates of 93%, 86%, and 80%, respectively. Fifty specimens in this study failed in the end to obtain DNA barcodes. Complete coverage for three core barcodes with a 71% sequencing success rate was achieved for 858 individuals. All specimen details, including species identifiers, voucher information, GPS coordinates, elevation, collection information, accession number, corresponding images, and DNA barcode sequences, were uploaded to the BOLD systems to establish a DNA barcode reference library of Lomas plants in Peru (Fig. 2). It is publicly available as “ DNA barcode for Lomas plants in Peru (DS-PLOMAS, 10.5883/DS-PLOMAS)”. In the BOLD systems, each record in a project represents a voucher specimen with its images, voucher collection numbers, associated sequences, and collection data associated with (1) Lab Sheet: Project Code, Process ID, Sample ID, Field ID, Catalog Num, ITS2 Seq. Length, ITS2 Trace Count, *rbcL* Seq. Length, *rbcL* Trace Count, *matK* Seq. Length, *matK* Trace Count, Image Count, Barcode Compliant, Stop Codon, Collection Date, Identification, Institution; (2) Voucher Info: Sample ID, Field ID, Museum ID, Institution Storing; (3) Taxonomy: Phylum, Class, Order, Family, Subfamily, Genus, Species, Identifier, Taxonomy Notes; (4) Collection Data: Collectors, Collection Date, Country/Ocean, State/Province, Region, Sector, Exact Site, GPS Coordinates, Elevation, and Collection Notes. All DNA barcodes are available in GenBank, with accession numbers OQ391231-OQ392357 for *rbcL*, OQ390168-OQ391205 for *matK*, and OQ411630-OQ412593 for ITS2 (Supplementary File 2). All Supplementary Files for this study are stored at Figshare^48^.

## Technical Validation

For this project, we conducted a review of all voucher specimens and repeated experiments for sequences that were identified as abnormal in BLASTn and Tree-based methods (Fig. 2). The results revealed 19 specimens that were inconsistent with morphological identification, and of the 12 specimens not identified to genus, eleven could be inferred as possible genera (Supplementary File 2). To ensure accuracy, these samples were re-sampled and sequenced, and the results were sent back to collaborating taxonomists for rechecking the specimens. The validated results have been noted in the corresponding specimens of the BOLD system, and the original records are retained for verification.

We evaluated the species identification ability of DNA barcodes using 125 species with multiple individuals (a total of 515 samples covering 125 species, 88 genera, and 34 families), and the results are shown in Table 4. The three-barcode combination (RMI2) had the highest correct identification capacity, ranging from 80% (BCM method) to 72% (Barcoding gap method), which is similar to results in previous DNA barcode studies (i.e., Fazekas *et al*.^49^; Hollingsworth *et al*.^50^; Naciri & Linder^51^; Wirta *et al*.^52^; Yan *et al*.^53^). Additionally, although 39% of the nodes in the 858-sample RMI2-ML tree (The RMI2-ML tree is available in Figshare^48^) had weak bootstrap support values (BS < 50%), there are still 15% of nodes with moderate support (BS from 50% to 70%), and 45% with high support (BS > 70%), which were sufficient to discriminate most genera and families. Of the 134 genera with multiple individuals, 17 (13%) were detected as not monophyletic (Supplementary File 2). These non-monophyletic relationships are partially due to unclear inter-generic boundaries (e.g., *Nassella* vs. *Jarava* and *Poa* vs. *Rostraria* in Poaceae, *Cyclanthera* vs. *Sicyos* in Cucurbitaceae, etc.), and insufficient barcode discrimination (e.g., *Convolvulus* in Convolvulaceae, *Villanova* in Asteraceae, *Croton* in Euphorbiaceae, etc.), and some were mixed with single individuals. At the family level, two of the 47 families with multiple individuals (4%) were not clustered into monophyletic groups (Asparagaceae and Amaryllidaceae).

**Table 4:** Species discrimination rates for core barcodes based on three methods for Peruvian Lomas plant.

Barcode type	BCM (taxonDNA)			Barcoding gap (K2P-distance)	Tree-based
	Correct identifications	Ambiguous	Incorrect identifications	with gap (%)	species identification (%)
*rbcL*	67%	30%	3%	66%	56%
*matK*	69%	27%	4%	67%	69%
ITS2	75%	21%	4%	74%	71%
RMI2^a^	80%	7%	13%	72%	76%

^a^combined *rbcL*, *matK*, and ITS2.

## Usage Notes

We provide freely accessible and downloadable historical plant lists and a DNA barcode reference library of Peruvian Lomas plants. Data as of the publication date are available for indexed search and free download from BOLD and GenBank. This library is anticipated to be of benefit to a wide range of applications, from species taxonomic revisions to specimen identification, including biogeographic and evolutionary studies in the region, to biodiversity surveys and monitoring, and environmental assessments.

## Data Availability

The code used to check species names and taxonomic authorities is available in the R package ‘plantlist’ version 0.8.0 (Zhang^28^).
